# Sustainable eco-friendly ratio-based spectrophotometric and HPTLC-densitometric methods for simultaneous analysis of co-formulated anti-migraine drugs with overlapped spectra

**DOI:** 10.1186/s13065-023-01020-2

**Published:** 2023-08-17

**Authors:** Christine Maged El-Maraghy

**Affiliations:** Analytical Chemistry department, Faculty of Pharmacy, October University for Modern Sciences and Arts (MSA), 6th October city, 11787 Egypt

**Keywords:** Aspirin, Metoclopramide, Spectrophotometry, HPTLC-densitometry, Green, GAPI, AGREE

## Abstract

Considering the green chemistry perspective and improving the environmental impact of quality control labs; two direct techniques with less hazardous solvents, less waste production and less energy consumption were developed for simultaneous analysis of Aspirin and Metoclopramide in bulk powder and pharmaceutical formulation. The ratio between the two drugs in their co-formulated preparation is very challenging; (90: 1, Aspirin: Metoclopramide). The first technique is spectrophotometry using simple mathematical operations; ratio difference and derivative ratio–zero crossing. The second technique is high-performance thin-layer chromatography (HPTLC) -densitometry which used a mobile phase consisting of cyclo-hexane: methanol: methylene chloride in a ratio of (1:4:1, v/v/v). The greenest solvents which give acceptable resolution were chosen. Following the International Conference on Harmonization (ICH) guidelines, the methods were found to be accurate, precise, and selective. Those methods were statistically compared to the reported spectrophotometric method and the results proved that there is no significant difference in accuracy and precision. Furthermore, the developed methods were assessed using the Analytical Eco-scale, Green Analytical Procedure Index (GAPI) and the Analytical Greenness calculator (AGREE), which gave a full image about their greenness profile. The spectrophotometry was found to be an excellent green technique compared to HPTLC with was considered an acceptable green one. The developed HPTLC-densitometric method was used for the first time for the analysis of this binary mixture. The two proposed spectrophotometric methos have advantages over the published methods as they used easy manipulation steps and are applied on the market pharmaceutical formulation. Owing to the advantages of the developed techniques; being green, do not require expensive sophisticated equipment or large volume of solvents; they could be used for routine analysis in quality control aspects.

**Supplementary Information:**

The online version contains supplementary material available at 10.1186/s13065-023-01020-2.

## Introduction

Migramax^®^ oral powder is used for treatment of migraine symptoms; headache, nausea and vomiting. The powder contains two active ingredients; Aspirin (ASP) and Metoclopramide (MET) in a ratio of (90: 1), respectively. ASP; acetylsalicylic acid which has analgesic, anti-inflammatory, and antipyretic properties [1] was aimed to reduce the headache accompanied with migraine. MET is a dopamine receptor antagonist and used as an antiemetic agent [2]. The chemical structural of MET and ASP are shown in Additional file 1: Fig. 1SM. The critical difference in the ratio between the two co-formulated drugs made their simultaneous analysis challenging. The literature reveals several methods for the analysis of ASP as single or with other drugs by spectrophotometry [3–9] and by TLC-densitometry [10–12] and for MET determination; spectrophotometric methods [13–18], and TLC methods [19, 20]. For the binary mixture, there are two reported spectrophotometric methods; the first one used the first derivative spectrophotometry and applied on laboratory prepared tablets which did not consider the critical ratio between the two drugs in the market pharmaceutical formulation (90: 1, ASP: MET) [21] and the second used absorptivity centering technique with complicated multi-mathematical operations [22]. An HPLC [21] and spectrofluorimetric [23] methods were also reported for their simultaneous analysis. To the best of our knowledge, there is no reported HPTLC-densitometric method for their simultaneous determination. The aim of this work was to develop green, fast, and economic spectrophotometric and HPTLC-densitometric methods for determination of ASP and MET in their co-formulated pharmaceutical preparation (Migramax^®^ oral powder) without interference from the excipients. The determination of the minute concentration of MET in presence of high concentration of ASP was our main challenge. The privilege of the developed spectrophotometric methods is the simplicity of the manipulation technique and its eco friendliness. The HPTLC-densitometric method has the advantages over the HPLC of being cheap as it does not require complicated programs nor large volume of solvents, and adjusting several conditions as the pH, temperature, and flow rate. So, the two techniques, the spectrophotometry and the HPTLC- densitometry, are adaptable techniques for quality control aspects. The developed methods were found to be green when assessed using three tools; the Analytical Eco-scale, Green Analytical Procedure Index (GAPI) and the Analytical Greenness calculator (AGREE).

## Experimental

### Apparatus and software

UV 1800 double beam UV–Visible spectrophotometer with UV-Probe software (V. 2.32, Shimadzu, Kyoto, Japan) was used for spectrophotometric analysis. HPTLC-densitometry was performed using Camag TLC scanner (Muttenz, Switzerland) operated with winCATS software (V. 3.15, Camag) and Camag Linomat ΙV autosampler (Muttenz, Switzerland). Aluminum TLC plates (20 × 20 cm) coated with 0.2 mm layer of silica gel F_254_ (Merck, Darmstadt, Germany). For the green assessment, AGREE free software (Provider: Universidadevago) was used.

### Materials and reagents

ASP and MET raw materials are obtained as kind gifts from Rameda Pharma (6th October city, Giza, Egypt) and Sunny Pharmaceutical (Badr city, Cairo, Egypt). The purities of ASP and MET were found to be 99.58 ± 0.56 and 99.78 ± 0.73, respectively as per the British pharmacopeia [24]. The solvents used; methanol (Merck, Darmstadt, Germany), cyclo-hexane and methylene chloride (ADWIC, Abu-Zaabal city, Qalyubia, Egypt).

Pharmaceutical formulation: Migramax^®^ 900/10 mg powder for oral solution (BN: 6M0006) (Zentiva company, United Kingdom). Each sachet was labeled to contain 1620 mg lysine acetylsalicylate equivalent to 900 mg acetylsalicylic acid and 10 mg metoclopramide hydrochloride.

### Standard solutions

ASP and MET stock solutions were prepared in concentration of (1 mg/mL) using methanol. Working solutions of both drugs were prepared by dilution from the corresponding stock solution to obtain concentration of (100 µg/mL) using the same solvent.

### Laboratory prepared mixtures

Five mixtures of ASP and MET were prepared by accurately transferring aliquots from both working solutions. The ratios of (ASP: MET) in the mixtures are: (90:1), (180:2), (150:10), (135:5), and (120: 10), respectively. The first two ratios mimic the ratio of the pharmaceutical formulation.

### Pharmaceutical formulation

Five packs of Migramax^®^ powder were mixed well and 0.177 mg of powder sachets (equivalent to 900 mg ASP and 10 mg MET) was weighed and sonicated for 30 min with 80 mL methanol and the solution was filtrated into 100 mL volumetric flask. A working solution was prepared by transferring 1 mL from the previously prepared solution into 100 mL volumetric flask and completed to the mark with methanol to obtain concentration of (ASP 90 µg/mL and MET 1 µg/mL). The working solution was analyzed, following the previously developed methods. The standard addition technique was conducted by spiking different concentrations of each of the two pure drugs to the pharmaceutical formulation in order to obtain final concentration within the linearity range of each drug and proceeding as the mentioned methods.

## Procedures

### Scanning by spectrophotometry

ASP and MET aliquots transferred from their corresponding working solutions (100 µg/mL) into two separate sets of 10 mL volumetric flasks, and then volume was completed with methanol to prepare concentrations of (15–200 µg/mL) for ASP and (1–40 µg/mL) for MET. The zero order absorption spectra were scanned in the range (200–400 nm) using methanol as blank.

### Optimized conditions of HPTLC-densitometry

The analysis of HPTLC-densitometry was performed on silica TLC plates. 10 µL of ASP and MET samples were applied as bands of 6 mm width using a micro-syringe in triplicate on the plates. The bands were applied 20 mm apart from the bottom line and 15 mm away from each other. The development of the bands was done using mobile phase consisting of cyclo-hexane: methanol: methylene chloride (1:4:1, v/v/v). The TLC chamber was left for 20 min for saturation with the mobile phase before the development process. The UV detection was performed at 270 nm.

### Construction of calibration curves

#### Spectrophotometric methods

For determination of ASP; a concentration of MET (20 µg/mL) was chosen as a divisor for the absorption spectra of ASP in the concentration range (15–200 µg/mL). A 20 µg/mL of ASP was the divisor for the absorption spectra of MET concentrations (1–40 µg/mL). The resulting ratio spectra were smoothed at Δλ = 10 nm.

*For the ratio difference method (RD)*, two wavelengths were selected from the ratio of the absorption spectra of ASP and MET respectively as to give the best linearity of the ratio spectra calibration curve. The difference in peak amplitudes between the two selected wavelengths 250 and 284 nm for ASP and 298 and 314 nm for MET were calculated. Calibration curves between the differences in the peak amplitudes versus the corresponding concentrations of each drug were constructed.

*For the Derivative ratio–zero crossing (DRZC),* the first derivative of the ratio spectra for both drugs was manipulated with intervals of Δλ = 10 nm and scaling factor of 10. The zero-crossing wavelength was chosen for measurement for each drug. The concentrations of ASP were proportional to the first derivative ratio signals at 255.5 nm (zero-crossing point with D^1^ of MET) and for the MET determination, the zero-crossing point was at 314 nm. The Calibration curves were obtained by measuring the first derivative ratio amplitudes at 255.5 nm and at 314 nm against the concentrations of ASP and MET, respectively.

#### HPTLC-densitometry

Aliquots transferred accurately from the working standard solutions of ASP and MET were separately applied in triplicate onto HPTLC plates to obtain final concentration range of (10–200 µg/band) for ASP and (1–45 µg/band) for MET. The plates were developed using the specified mobile phase. The bands were scanned at 270 nm. The calibration curves were constructed by plotting the average peak area of the bands against the corresponding concentration of each drug, from which the regression equations were calculated.

### Application to laboratory prepared mixtures

Concerning the spectrophotometric methods, the ratio spectra of the laboratory prepared mixtures were calculated as previously mentioned using ASP and MET as divisors for determination of MET and ASP, respectively. The developed two methods were applied on the mixtures and the absorbance was measured at the previously specified wavelengths. The recovery % of the drugs was calculated using the corresponding regression equation for each method.

Concerning the HPTLC-densitometry, the laboratory mixtures were applied as bands on the plates in triplicate. The concentration of ASP and MET were calculated using the corresponding regression equation.

### Application to pharmaceutical formulation

The developed procedures were applied for analysis of ASP and MET in the prepared pharmaceutical formulation (Migramax^®^ oral powder). The concentrations of both drugs were determined using the corresponding computed regression equations.

## Results and discussion

The quality control analysts preferred the use of green, rapid, cheap, and reproducible methods for the analysis of repeated pharmaceutical batches. So, we choose to develop two techniques: spectrophotometric methods [ratio difference (RD) and the Derivative ratio–zero crossing (DRZC)] which do not require complicated calculation and manipulation and the HPTLC- densitometric method which has the advantages of small volume of solvents used, low cost and does not use sophisticated equipment when compared to the commonly used HPLC method. These developed methods fulfill the requirements of quality control analysts. Our studied binary mixture is ASP and MET which are co-formulated in a challenging ratio of (90:1, respectively).

### Spectrophotometric methods

**Fig. 1:**
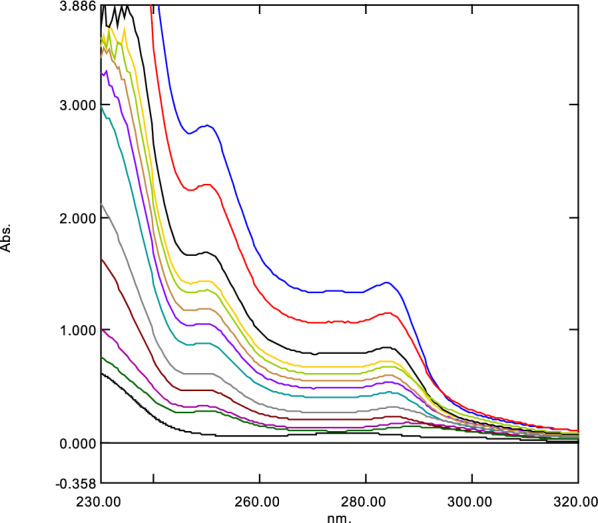
Ratio spectra of different concentration ASP (15–200 μg/mL) using 20 μg/mL of MET as a divisor

**Fig. 2:**
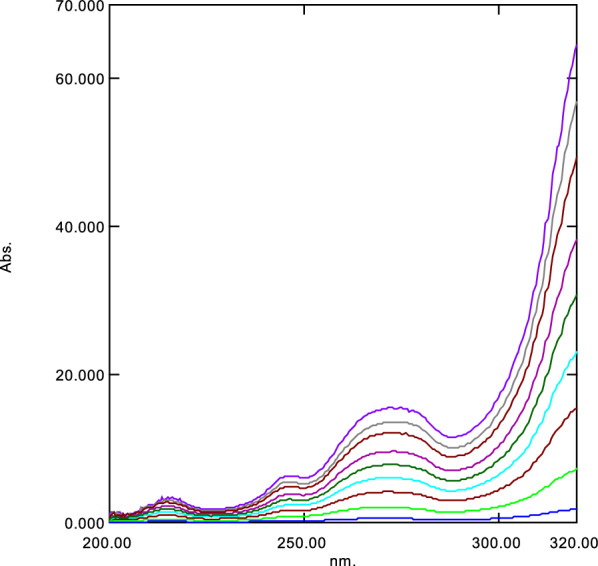
Ratio spectra of different concentration MET (1–40 μg/mL) using 20 μg/mL of ASP as divisor

The zero order absorption spectra of ASP and MET show a severe overlapping, Additional file 1: Fig. 2SM. So, the direct method of using the zero order spectra could not be applied for this binary mixture. The two developed methods (RD) and (DRZC) have the advantages of simple two- step manipulations (so signal to noise ratio was enhanced). The two methods are well-developed, accurate and precise methods [25–27]. For the construction of the ratio spectra, (Figs. 1 and 2); the divisor concentration was chosen as to give the minimum noise and highest sensitivity [28]. In the RD method, the two wavelengths were selected to obtain the optimum linearity of the calibration curve [29] and were chosen so as; for determination of ASP, the two wavelengths 250 and 284 nm were selected, at which the ratio spectra of MET show the same absorbance whereas ASP ratio spectra shows significant difference in absorbance values. Similarly, the wavelengths 298 and 314 nm were selected for the analysis of MET. This method has a main privilege that the difference in absorbance between the two selected wavelengths of the ratio spectra is directly proportional to the concentration of the component of interest; does not depend on the other interfering component. Additionally, this method has an advantage over DRZC method; the elimination of the step of derivative calculation and consequently the signal to noise ratio is enhanced.

**Fig. 3:**
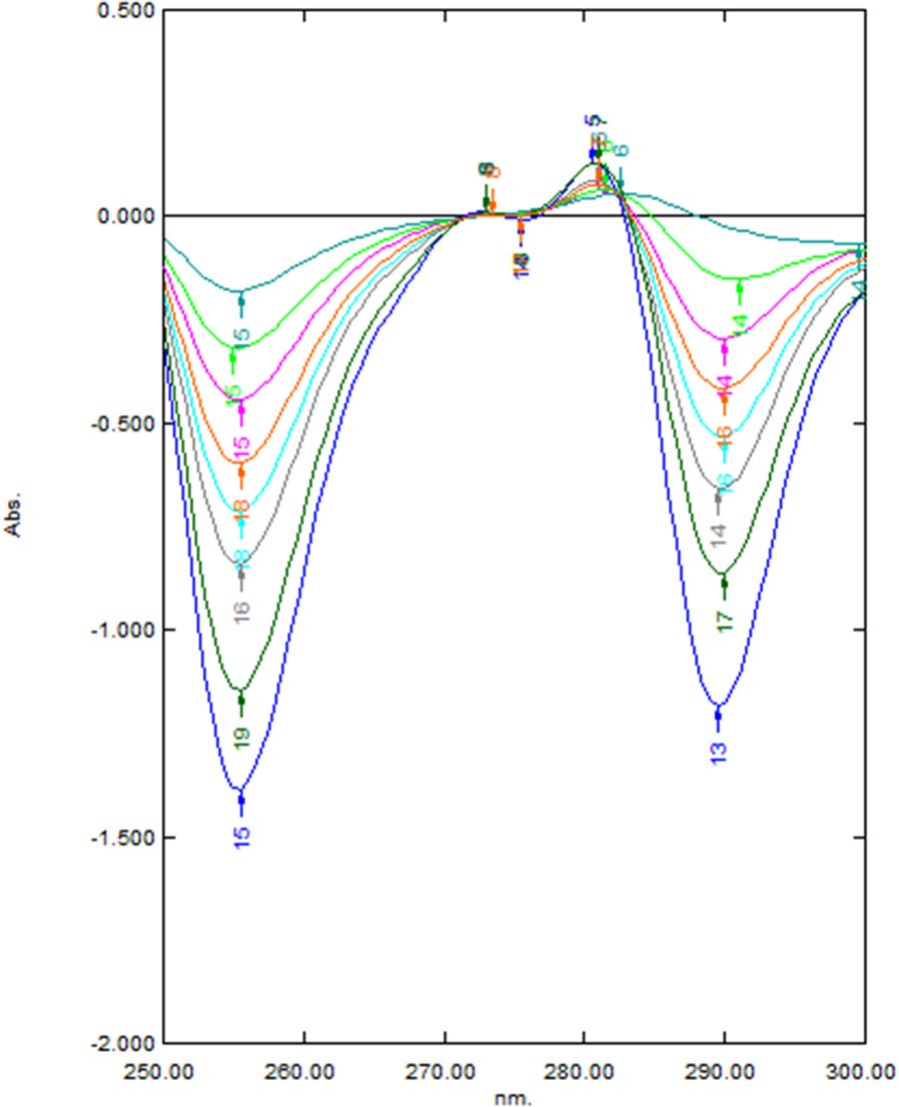
First derivative ratio spectra of different concentration of ASP (15–200 μg/mL) at 255.5 nm, using 20 μg/mL of MET as a divisor

**Fig. 4:**
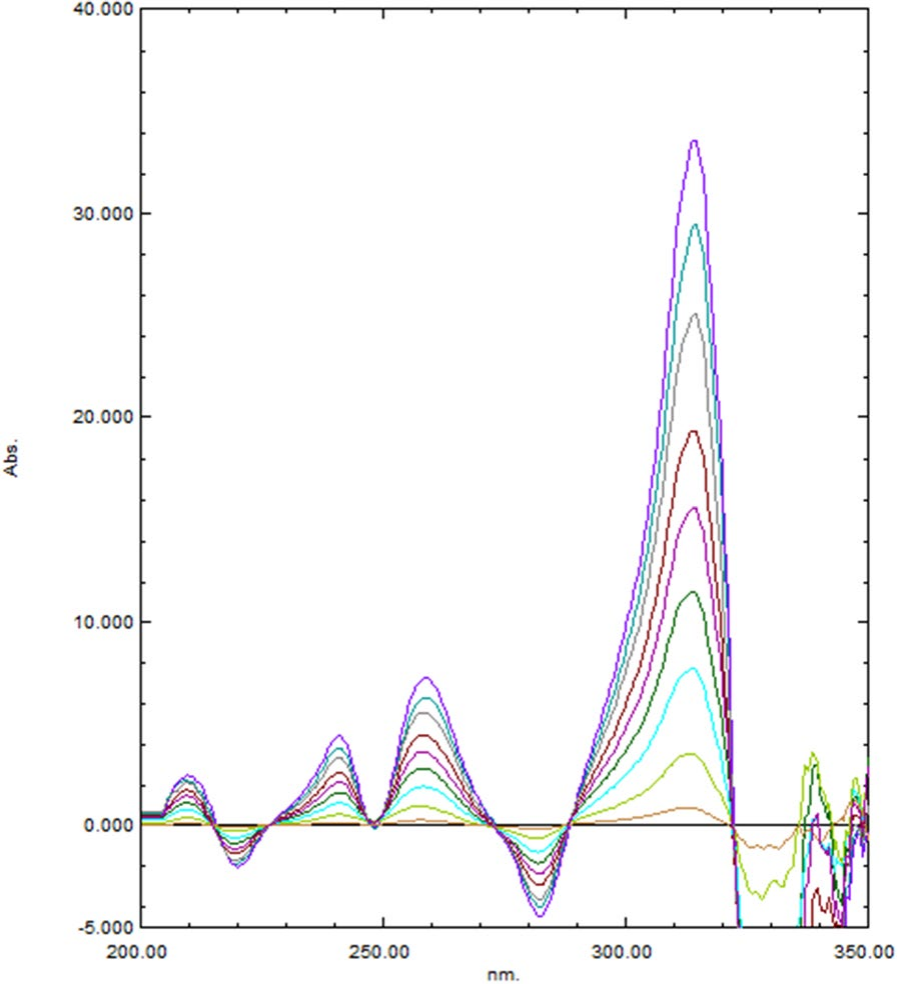
First derivative ratio spectra of different concentration MET (1–40 μg/mL) at 314 nm, using 20 μg/mL of ASP as a divisor

For the DRZC; the first derivative of the ratio spectra of both drugs was manipulated and the drug was measured at the zero-crossing point of the other drug to eliminate its interference in the binary mixture. The first derivative was generated with intervals of Δλ = 10 nm. The concentrations of ASP in the binary mixtures were determined by measuring the amplitudes of first derivative spectra of the ratio spectra at 255.5 nm (zero-crossing of D^1^ of MET) using the corresponding regression equation as demonstrated in Fig. 3. The same was proceeded for the analysis of MET at 314 nm (zero-crossing of D^1^ of ASP), Fig. 4.

### HPTLC-densitometry

**Fig. 5:**
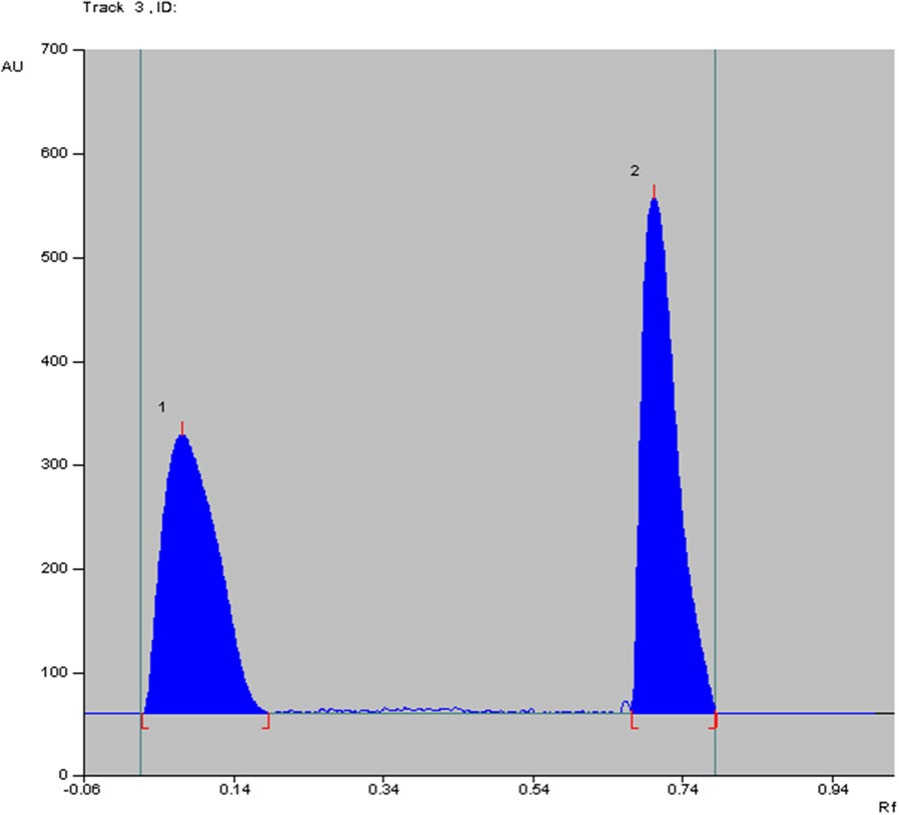
^2^D HPTLC densitogram of the resolved mixture of MET (2.0 μg/ band) at R_f_ = 0.7 ± 0.02 and ASP (26.0 μg/ band) at R_f_ = 0.08 ± 0.03, using cyclo-hexane: methanol: methylene chloride (1:4:1, v/v/v) with UV detection at 270 nm

**Fig. 6:**
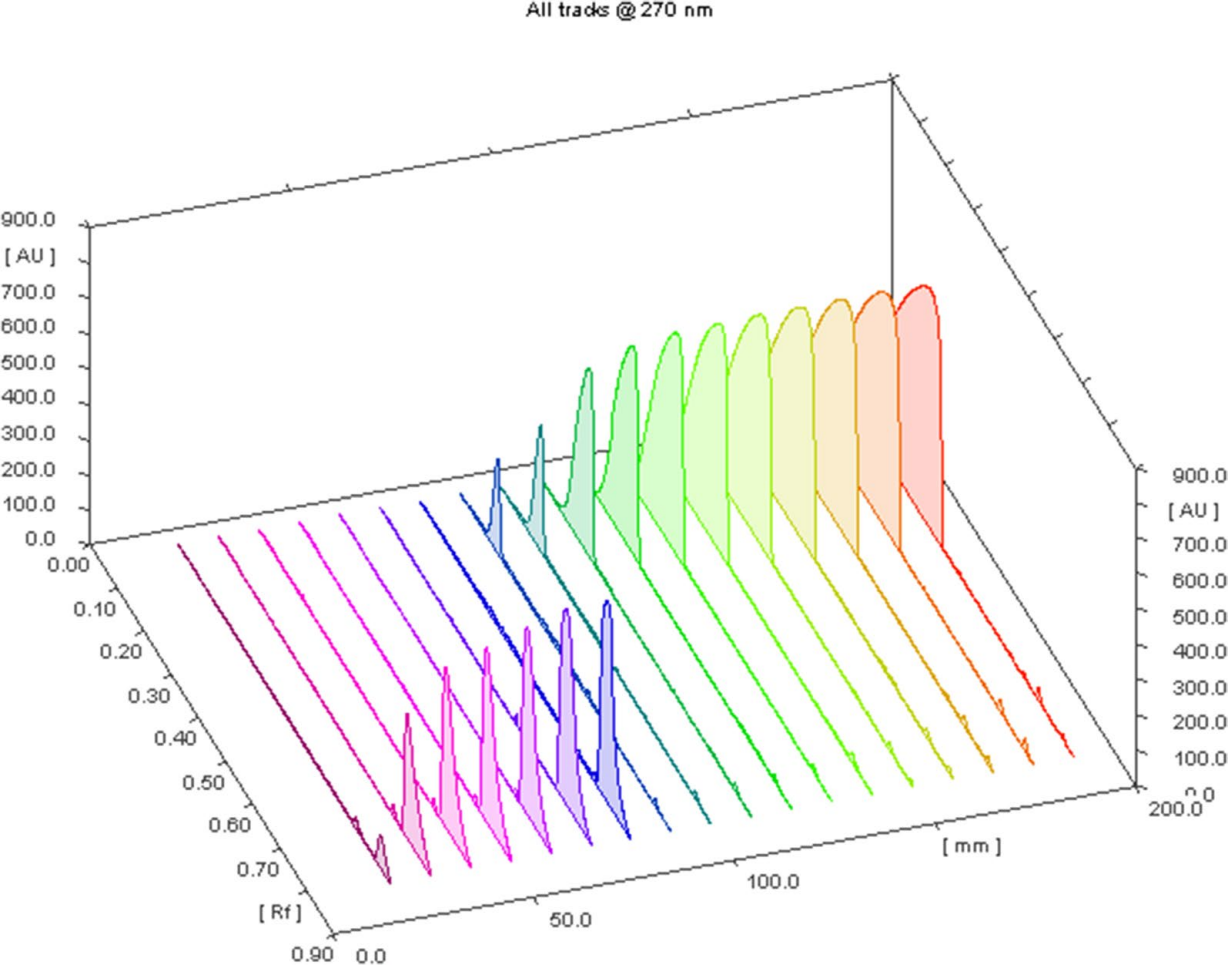
^3^D HPTLC- densitogram of ASP (10–200 µg/band) and MET (1- 45 µg/band), using cyclo-hexane: methanol: methylene chloride (1:4:1, v/v/v) with UV detection at 270 nm

The principle of the TLC is that the separation between the compounds depends on the difference in their retardation factor (R_f_) which consequently depends on the difference in their polarities and their migration rates on the TLC plates. TLC was used for simultaneous analysis of drug with its related compounds/or impurities [30, 31]. The chromatographic conditions were optimized by trying different green solvent mixtures to achieve optimum separation and resolution. Initially, a mixture of methanol and ethyl acetate; commonly used green solvents; was tried in different ratios but no separation was resulted and the drugs which are highly polar moved with the polar mobile phase (methanol polarity index = 5.1 and ethyl acetate polarity index = 4.4) till the solvent front. We tried less polar green solvents (1- butanol and 1-propanol) but the dugs did not move more than 3 cm from the bottom line. We tried methanol: cyclo- hexane mixture to decrease the system polarity-the cyclo-hexane is in the amber region of GlaxoSmithKline (GSK) solvent guide [32]. The resolution was less than two units using different ratios of methanol and cyclo-hexane. So, we added to the methanol and cyclo-hexane mixture, the methylene chloride (of medium polarity; polarity index = 3.1) to move the two drugs to more than the half of the plate height. We tried different ratios of these three solvents. Finally, a system of cyclo-hexane: methanol: methylene chloride in a ratio of (1:4:1, v/v/v) could achieve the best greenness profile and optimum resolution between ASP and MET, Fig. 5. The ^3^D HPTLC-densitogram linearity range of both drugs was shown in Fig. 6**.**

### Method validation

The validation parameters of the spectrophotometric and HPTLC- densitometric methods were checked as per the ICH guidelines [33]. The results of linearity range, accuracy, precision, LOD and LOQ were listed in Table 1. The linearity range for the HPTLC- densitometry was 10–200 µg/band for ASP and 1–45 µg/band for MET and for the spectrophotometry; 15–200 µg/mL for ASP and 1–40 µg/mL for MET. The accuracy was presented in term of recovery % for each drug separately which was measured from the corresponding regression equation and the recovery range was from 98.36 to 100.12%. The intra-day and inter-day precision results were accepted and the RSD% values were less than 2 units. The specificity was assessed by measuring the recovery of each drug in five laboratory prepared mixtures; good results were obtained which confirm that the methods are selective, Table 2.

**Table 1:** The regression parameters and validation results for determination of ASP and MET by the developed methods

Parameters	HPTLC-densitometry		Spectrophotometric methods			
			RD		DRZC	
	ASP	MET	ASP	MET	ASP	MET
Linearity (µg/band or µg/mL)	10–200	1–45	15–200	1.0–40	15–200	1.0–40
Slope	71.490	2871.3	0.006894	0.7983	0.006750	0.8559
Intercept	15129	4731.1	0.02435	− 0.3013	0.04388	− 0.9234
Correlation Coefficient(r)	0.9996	0.9996	0.9997	0.9994	0.9993	0.9993
Accuracy ^ab^(Recovery% ± SD)	98.53 ± 1.47	98.36 ± 1.25	100.12 ± 1.36	99.54 ± 0.95	99.67 ± 1.68	100.20 ± 0.58
LOD (µg/band or µg/mL)	3.607	0.057	4.25	0.26	5.41	0.18
LOQ (µg/band or µg/mL)	9.02	0.76	14.9	0.93	14.4	0.73
Intra-day precision ^ac^RSD%	1.75	1.26	0.39	0.46	0.76	1.23
Inter-day precision ^ac^RSD%	1.96	1.63	0.50	1.05	0.98	1.70

^a^Average of three experiments

^b^(n = 5 concentrations; for ASP (15,30,90,130,170 µg/band or µg/mL) and for MET (3,9,15,25,35 µg/band or µg/mL)

^c^(n = 3 concentrations; for ASP (50,100,150 µg/band or µg/mL) and for MET (5, 15, 30 µg/band or µg/mL)

**Table 2:** Experimental results for the analysis of laboratory prepared mixtures using the developed methods

Laboratory prepared mixtures (µg/band or µg/mL)		HPTLC-densitometry		Spectrophotometric methods			
				RD		DRZC	
		Recovery%^a^					
ASP	MET	ASP	MET	ASP	MET	ASP	MET
^b^90	1	97.56	98.40	100.45	100.32	99.74	98.14
^b^180	2	99.12	99.46	100.37	101.51	98.35	99.54
150	10	100.54	100.45	99.56	100.76	99.57	100.76
135	5	98.43	100.78	99.41	100.33	100.38	99.36
120	10	100.32	96.09	100.47	99.78	100.94	99.58
Mean recovery %		99.19	99.03	100.05	100.54	99.79	99.47
± SD		1.25	1.89	0.52	0.64	0.97	0.93

^a^Average of three experiments

^b^Ratio present in the pharmaceutical dosage form (Migarmax^®^ oral powder)

### System suitability for the HPTLC- densitometry

The system suitability was tested to confirm that the system functions correctly as per USP [34]. The values of retardation factor (R_f_), resolution (R_s_), tailing factor (T) and selectivity factor (α) were calculated and listed in Table 3.

**Table 3:** System suitability parameters of the developed HPTLC–densitometric method

Parameters	ASP	MET	Reference values [34]
R_f_	0.08 ± 0.03	0.7 ± 0.02	
Tailing factor (T)	1.10	1.43	~ 1
Selectivity factor (α)	7.34	–	> 1
Resolution (R_S_)	5.52	–	> 1.5

T = W0.05/2f, where W0.05 is the width of the peak at 5% height and f is the distance from peak maximum to the leading edge of peak

α = k´2/k´1, where k´ is the capacity factor; k´ = (1–R_f_)/R_f_

Rs = [2 (R_f2_–R_f1_)]/(W1 + W2), Where R_f_ is retardation factor and W is peak width

### Application of the developed methods to pharmaceutical formulation

**Fig. 7:**
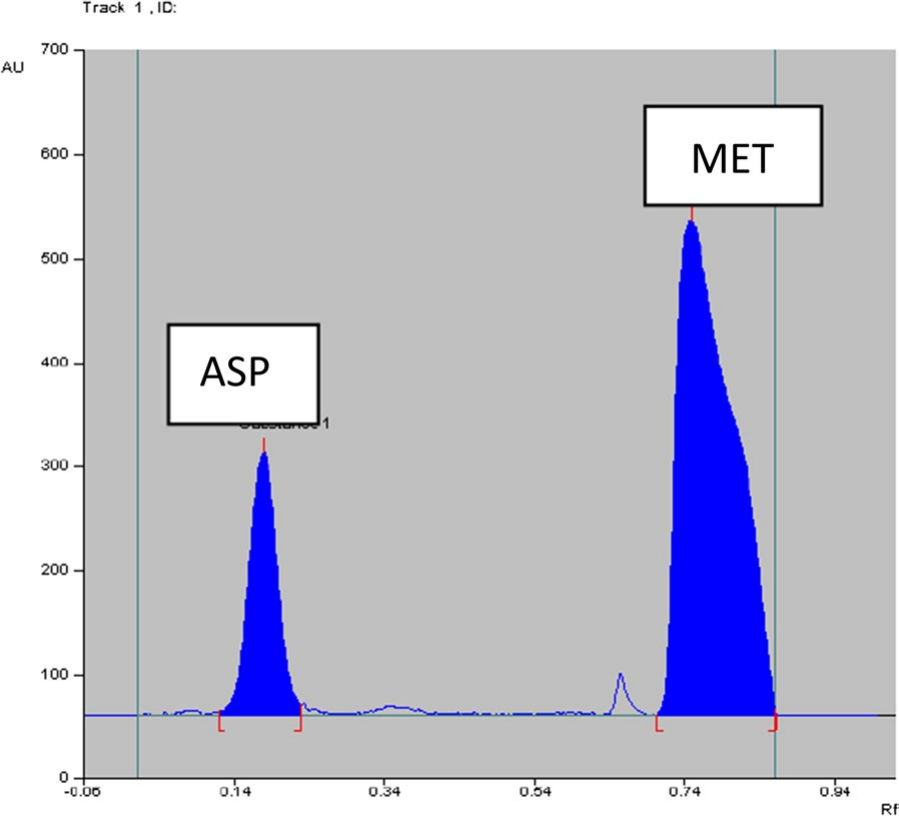
^2^D HPTLC-densitogram of co-formulated ASP and MET in Migramax^®^ oral powder, using cyclo-hexane: methanol: methylene chloride (1:4:1, v/v/v) with UV detection at 270 nm

The three developed methods were applied successfully to Migramax^®^ with challenging ratio between ASP and MET (90:1). The methods were able to determine each drug without interference from excipients, as shown in Fig. 7. As a recommendation, the minor peak appearing in Fig. 7 could be identified using TLC/MS, as it may be due to the solvent used. The standard addition technique was conducted to assure the validity of the methods, as shown in Table 4.

**Table 4:** Application of the proposed methods for the analysis of pharmaceutical formulation and results obtained by standard addition technique

^a^Mean Recovery % ± SD		HPTLC-densitometry		RD		DRZC	
		ASP	MET	ASP	MET	ASP	MET
Migaramx^®^ oral powder claimed to contain 900 mg ASP and 10 mg MET		99.16 ± 1.27	101.43 ± 1.82	100.74 ± 0.48	100.41 ± 0.88	99.55 ± 1.26	99.81 ± 0.59
Standard addition technique							
Pure added (µg/band or µg/mL)		^b^Mean recovery % ± SD					
ASP	MET						
20	5	100.43 ± 1.47	101.76 ± 0.64	99.65 ± 0.64	99.54 ± 0.95	100.41 ± 0.88	99.81 ± 0.59
40	20	99.52 ± 1.23	100.32 ± 1.31	100.83 ± 0.73	100.79 ± 0.53	100.05 ± 1.32	99.53 ± 0.65
60	30	100.82 ± 0.58	100.46 ± 1.25	100.72 ± 1.02	100.32 ± 0.76	100.31 ± 0.85	100.64 ± 0.83

^a^Average of six experiments

^b^Average of three experiments

### Statistical analysis

The developed methods were compared statically to the published multi- steps calculation spectrophotometric method [22]. The data in Table 5 showed that there is no significant difference in terms of accuracy and precision between the developed and the published methods.

**Table 5:** Statistical comparison for the results obtained by the proposed methods and the reported method for the analysis of ASP and MET in pure powder form

Values	ASP			MET			^a^Reported method [22]
	HPTLC	RD	DRZC	HPTLC	RD	DRZC	
Mean	99.16	100.74	99.55	101.43	100.41	99.81	99.76
SD	1.27	0.48	1.26	1.82	0.88	0.59	0.88
n	6	6	6	6	6	6	5
Variance	1.61	0.23	1.58	3.31	0.77	0.34	0.77
^b^Student’s *t*-test	0.426 (2.306)	1.47 (2.26)	1.99 (2.26)	1.583 (2.306)	0.105 (2.145)	0.401 (2.145)	–
^b^*F* –value	1.613 (6.400)	3.497 (6.094)	2.943 (6.094)	1.158 (6.400)	1.486 (3.478)	1.486 (3.478)	–

^a^The factorized spectrum method for determination of ASP and MET

^b^The values between parenthesis are the theoretical values of *t*- and *F*-test at *P* = 0.05

### Evaluation of the green character of the developed methods

While developing our methods, we took into consideration the environmental protection issues during solvent selection, the selection of the analytical method, the volume of waste and the waste disposal. For the measurement of the green character, we applied three tools of assessment- The Analytical Eco-scale [35], Green Analytical Procedure Index (GAPI) [36] and the Analytical Greenness calculator (AGREE) [37]. The use of more than one tool of assessment is preferable when comparing analytical methods [38–40]. The Analytical Eco-scale is a semi-quantitative tool based on giving penalty points to the parameters which are not considered eco-friendly. Table 6 showed that the analytical Eco-Scale score of the spectrophotometric method is higher than that of HPTLC; as the last method used cyclohexane and methylene chloride which increase its penalty points. The reagents hazards are based on the Globally Harmonized System of Classification and Labeling of Chemicals (GHS) [41]. The GAPI tool has advantages over the Analytical Eco-scale as it assesses the whole analytical procedure, from sampling to final determination and takes into consideration the safety and health hazards of the reagents used. GAPI provides qualitative information as pictogram symbol, shown in Table 6. Additional file 1: Table 1SM showed the 15 points of comparison between the two developed methods which are identical except for field 10. The spectrophotometry has field 10 greenly shaded field but HPTLC has this field yellow because of the health hazard of methylene chloride which is ranked 2 based on the solvent selection guides [42, 43]. For the newly launched AGREE tool, it is a calculator using freely available software that allows rapid, easy, and comprehensive assessment for the green character. It is presented as a final score in the middle of the pictogram depending on the fulfillment of 12 principles of green analytical chemistry. The figures of AGREE have score of 0.74 and 0.71 for the spectrophotometric and HPTLC-densitometric methods, respectively, as shown in Table 6. In conclusion, the spectrophotometric method was greener than the HPTLC developed method.

**Table 6:** Assessment of the green character for the developed methods using Eco-scale, GAPI and AGREE tools

Parameters	HPTLC-densitometry		Spectrophotometric methods	
Preparation	Reagent used:	Penalty points (PPs)	Solvents used:	Penalty points (PPs)
	Methanol	12	Methanol	12
	Cyclo-hexane	8		
	methylene chloride	1		
Amount of reagents	> 100 mL	3	> 100 mL	3
Instrument	HPTLC scanner and autosampler (enery used < 0.1 kWh per sample)	0	UV–Vis Spectrometry (energy used < 0.1 kWh per sample)	0
Occupational hazards	Analytical process hermetization	0	Analytical process hermetization	0
Waste	The waste volume is 1–10 mL per sample	3	The waste volume is 1–10 mL per sample	3
Total penalty points	∑ 27		∑ 18	
Analytical Eco-Scale total score	73 Acceptable green		82 Excellent green	
GAPI				
AGREE				

## Conclusion

Two eco-friendly analytical methods were developed for determination of ASP and MET in their co-formulated preparation. The green analytical chemistry attributes were considered; spectrophotometry and HPTLC-densitometry have low energy consumption and use green less hazardous solvents. The developed methods were assessed by three tools; The Analytical Eco-scale, GAPI and AGREE, and found to be green. The spectrophotometric methods are easy to apply as they use simple mathematical operation. For the HPTLC-densitometric technique, it is cheap with no complicated conditions of development as for the reported HPLC method and it is the first time to be used for the analysis of this binary mixture. Both developed methods were found to be accurate, and selective according to ICH guidelines. Consequently, these methods could be applied for routine analysis of ASP and MET in their bulk powder and pharmaceutical formulation in quality control lab.

### Supplementary Information

**Additional file 1: Fig. 1SM.** Chemical structures of (a) ASP and (b) MET. **Fig. 2SM.** Zero order absorption spectra of ASP (-) (90 μg/mL) and MET (….)(1.0 μg/mL). **Table 1SM.** Green Analytical Procedure Index parameters (GAPI) for the proposed methods

## Data Availability

All data generated or analyzed during this study are included in this published article.

## Abbreviations

AGREE: Analytical greenness calculator
ASP: Aspirin
D1: First derivative
DRZC: Derivative ratio–zero crossing
GAPI: Green analytical procedure index
GSK: GlaxoSmithKline
HPTLC: High performance thin layer chromatography
ICH: International Conference on Harmonization
LOD: Limit of detection
LOQ: Limit of quantitation
MET: Metoclopramide
RD: Ratio difference
R_f_: Retardation factor
R_s_: Resolution
T: Tailing factor

## References

[1] Martindale (2013). The complete drug reference electronic version.

[2] Ritter J, Lewis L, Mant T, Ferro A (2008). A text book of clinical pharmacology and therapeutics.

[3] Moţ AC, Soponar F, Medvedovici A, Sârbu C (2010). Simultaneous spectrophotometric determination of aspirin, paracetamol, caffeine, and chlorphenamine from pharmaceutical formulations using multivariate regression methods. Anal Lett.

[4] Jones M, Thatcher RL (1951). Spectrophotometric determination of aspirin, phenacetin, and caffeine in mixtures. Anal Chem.

[5] Verma KK, Jain A (1986). Spectrophotometric determination of aspirin by trans acetylation of 4-aminophenol. Anal Chem.

[6] Albakaa ARM, Ahmed MA, Mohammed BT, Jabbar ZA (2019). Development method for determination of aspirin as sodium salicylate by UV-VIS spectroscopy. IOP Conf Ser Mater Sci Eng.

[7] Ali NW, Abdelwahab NS, Abdelkawy M, Emam AA (2012). Validated spectrophotometric and spectrodensitometric methods for determination of a ternary mixture of analgesic drugs in different dosage forms. Bull Fac Pharm Cairo Univ.

[8] Glombitza BW, Schmidt PC (1994). Comparison of three new spectrophotometric methods for simultaneous determination of aspirin and salicylic acid in tablets without separation of pharmaceutical excipients. J Pharm Sci.

[9] Hajian R, Soltaninezhad A (2013). The spectrophotometric multicomponent analysis of a ternary mixture of paracetamol, aspirin, and caffeine by the double divisor-ratio spectra derivative method. J Spectrosc.

[10] Bocheńska P, Pyka A (2012). Determination of acetylsalicylic acid in pharmaceutical drugs by tlc with densitometric detection in UV. J Liq Chromatogr Relat Technol.

[11] Bhusari VK, Dhaneshwar SR (2012). Validated HPTLC method for simultaneous estimation of atenolol and aspirin in bulk drug and formulation. Anal Chem.

[12] Pyka-Pajak A, Dolowy M, Parys W, Bober K, Janikowska Grazyna (2018). A simple and cost-effective TLC-densitometric method for the quantitative determination of acetylsalicylic acid and ascorbic acid in combined effervescent tablets. Molecules.

[13] Naggar A, Elnasr T, Sayed AA (2017). Determination of metoclopramide hydrochloride in pharmaceutical formulations using three different spectrophotometric methods. Pharm Anal Acta.

[14] Shah J, Jan MR, Khan MA, Amin S (2005). Spectrophotometric determination of metoclopramide in pharmaceutical preparations. J Anal Chem.

[15] Devi OZ, Basavaiah K, Vinay KB, Revanasiddappa HD (2016). Sensitive spectrophotometric determination of metoclopramide hydrochloride in dosage forms and spiked human urine using vanillin. Arab J Chem.

[16] El-Gendy AE (1992). Spectrophotometric determination of metoclopramide VIA charge-transfer complexes. Spectrosc Lett.

[17] Basheer MY, Kashif AA, Aljaily A, Ibrahim MM, Osman HM (2017). Development and validation of UV-spectroscopic method for assay of metoclopramide hydrochloride in bulk and injectable dosage form. Am J Res Commun.

[18] Nassar MW, Attia KA, Abouserie AA, Said RA, Abdel-Kareem RF (2018). Comparative study on four UV spectrophotometric methods manipulating ratio spectra for the determination of metoclopramide monohydrochloride monohydrate in presence of its acidic degradate. J Anal Pharm Res.

[19] Hegazy AM, Hassan NY, Metwally FH, Abdel-Kawy M (2013). Application and validation of two smart spectrophotometric and a HP-TLC densitometric methods for determination of metoclopramide hydrochloride/paracetamol in raw material and in pharmaceuticals. Int J Pharm.

[20] Huizing G, Beckett AH (1979). Rapid thin-layer chromatographic method for the determination photodensitometric of metoclopramide and clebopride in the presence of some of their metabolic products. J Chromatogr.

[21] Belal FF, El-Din MKS, Tolba MM, Elmansi H (2014). Derivative spectrophotometric and liquid chromatographic methods for the simultaneous determination of metoclopramide hydrochloride and aspirin in pharmaceuticals. J Chromatogr Sci.

[22] Lotfy HM, Saleh SS, El-Maraghy CM (2020). Advanced approaches for the treatment and amplification of weak spectral signals produced by critical concentrations in white multicomponent systems. Spectrochim Acta A Mol Biomol Spectrosc.

[23] Elmansi H, El Abass Abo, Mohamed S, Fathy ME (2016). Simultaneous determination of metoclopramide and aspirin by spectrofluorimetric technique: application to pharmaceutical formulations and human plasma. Anal Methods.

[24] Elbalkiny HT, Yehia AM, Safa’a MR, Elsaharty YS (2019). Removal and tracing of cephalosporins in industrial wastewater by SPE-HPLC: optimization of adsorption kinetics on mesoporous silica nanoparticles. J Anal Sci Technol.

[25] El-Maraghy CM, Salem H, Amer SM, Nebsen M (2019). Stability indicating spectrophotometric and chemometric methods for determination of aripiprazole in presence of its degradation products, a comparative study. Anal Chem Lett.

[26] El-Maraghy CM, Mohamed EH (2018). Successive stability indicating spectrophotometric technique for simultaneous determination of quetiapine fumarate and its three major related compounds. Curr Anal Chem.

[27] Chv S, Gupta S, Chandan AK, Gunturu C, Indracanti M (2012). Determination of cefixime and ofloxacin by ratio spectra and zero crossing difference spectrophotometry. Int J Pharm Pharm Sci.

[28] El-Maraghy CM, Lamie NT (2019). Three smart spectrophotometric methods for resolution of severely overlapped binary mixture of Ibuprofen and paracetamol in pharmaceutical dosage form. BMC Chem.

[29] Elzanfaly ES, Saadn AS, Elaleem AEBA (2012). A smart simple spectrophotometric method for simultaneous determination of binary mixtures. J Pharm Anal.

[30] Salem H, Amer SM, Maraghy CME, Nebsen M (2015). Validated HPLC and thin layer-densitometric methods for determination of quetiapine fumarate in presence of its related compounds. J Chromatogr Sep Tech.

[31] El-Maraghy CM (2023). Implementation of green chemistry to develop HPLC/UV and HPTLC methods for the quality control of fluconazole in presence of two official impurities in drug substance and pharmaceutical formulations. Sustain Chem Pharm.

[32] Alder CM, Hayler JD, Henderson RK, Redman AM, Shukla L, Shuster LE (2016). Updating and further expanding GSK’s solvent sustainability guide. Geen Chem.

[33] Prat D, Hayler J, Wells A (2014). A survey of solvent selection guides. Green Chem.

[34] United States Pharmacopeia (2011). The official compendia of standards, USP 34-NF 29.

[35] Gałuszka A, Migaszewski ZM, Konieczka P, Namieśnik J (2012). Analytical eco-scale for assessing the greenness of analytical procedures. TrAC.

[36] Plotka-Wasylka J (2018). A new tool for the evaluation of the analytical procedure: green analytical procedure index. Talanta.

[37] Pena-Pereira F, Wojnowski W, Tobiszewski M (2020). AGREE-analytical GREEnness metric approach and software. Anal Chem.

[38] Prajapati PB, Radadiya K, Shah SA (2022). Quality risk management based: analytical quality by design approach to eco-friendly and versatile chromatography method for simultaneous estimation of multiple fixed-dose-combination products of anti-diabetic drugs. J Pharm Innov.

[39] Prajapati P, Shah H, Shah SA (2022). Implementation of QRM and DoE-based quality by design approach to VEER chromatography method for simultaneous estimation of multiple combined dosage forms of paracetamol. J Pharm Innov.

[40] Gamal M, Naguib IA, Panda DS, Abdallah FF (2021). Comparative study of four greenness assessment tools for selection of greenest analytical method for assay of hyoscine N-butyl bromide. Anal Methods.

[41] Globally harmonized system of classification and labelling of chemicals (GHS). 4th ed. New York: United Nations Economic Commission for Europe (UNECE); 2011.

[42] Prat D, AndyWells Hayler J, Sneddon H, McElroy CR, Abou-Shehadad S (2016). CHEM21 selection guide of classical- and less classical-solvents. Geen Chem.

[43] Tobiszewski M (2016). Metrics for green analytical chemistry. AnalMethods.

