# Supplementary material for: Sustainable eco-friendly ratio-based spectrophotometric and HPTLC-densitometric methods for simultaneous analysis of co-formulated anti-migraine drugs with overlapped spectra
Source: BMC Chem. 2023 Aug 17;17(1):100. doi: 10.1186/s13065-023-01020-2 (PMC10433579; doi:10.1186/s13065-023-01020-2)
Supplement: Supplementary file 1 — Additional file 1: Fig. 1SM. Chemical structures of (a) ASP and (b) MET. Fig. 2SM. Zero order absorption spectra of ASP (-) (90 μg/mL) and MET (….)(1.0 μg/mL). Table 1SM. Green Analytical Procedure Index parameters (GAPI) for the proposed methods [file 13065_2023_1020_MOESM1_ESM.docx]

**Additional file**


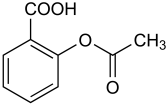
**
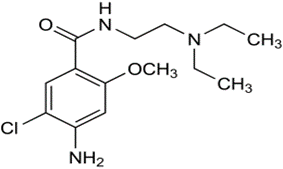
**

(a) (b)

**Fig.1SM.** Chemical structures of (a) ASP and (b) MET

**Fig.2SM.** Zero order absorption spectra of ASP (-) (90 μg/mL) and MET (….)(1.0 μg/mL)

**Table 1SM.** Green Analytical Procedure Index parameters (GAPI) for the proposed methods

| **Category** | **HPTLC-densitometry** | **Spectrophotometric methods** |
| --- | --- | --- |
| 1. **Collection** | *Off-line* | *Off-line* |
| 1. **Preservation** | None | None |
| 1. **Transport** | None | None |
| 1. **Storage** | None | None |
| 1. **Type of the method: direct/indirect** | Direct (no sample preparation) | Direct (no sample preparation) |
| 1. **Scale of extraction** | None | None |
| 1. **Solvents/reagents** | Solvent-free extraction method | Solvent-free extraction method |
| 1. **Additional treatments** | None | None |
| **Reagents and solvents** | | |
| 1. **Amounts** | 10-100 mL | 10-100 mL |
| 1. **Health hazard** | Methanol: ; Slightly toxic, slight irritant NFPA health hazard rating=1  Cyclo-hexane: Can cause significant irritation, NFPA health hazard rating=1  Methylene chloride: NFPA health hazard rating=2 | Methanol: NFPA health hazard rating=1 |
| 1. **Safety hazard** | Methanol:  :  instability score= 0, flammability score=3  Cyclo-hexane: :  instability score= 0, flammability score=3  Methylene chloride: instability score= 0, flammability score=1 | Methanol:  :  instability score= 0, flammability score=3, Normally stable, even under fire conditions |
| **Instrumentation** | | |
| 1. **Energy** | ≤0.1 kWh per sample | ≤0.1 kWh per sample |
| 1. **Occupational hazard** | Hermetic sealing of analytical process | Hermetic sealing of analytical process |
| 1. **Waste** | >10 mL | >10 mL |
| 1. ***Waste treatment** | treatment | treatment |
| Circle in the middle of GAPI: *Procedure for qualification and*  *quantification* | | |

NFPA: National Fire Protection Association

* The waste of the developed methods was collected and treated by “Lafarge Egypt for Waste management” company
